# A case of Kommerell diverticulum in adolescence presented with dysphagia

**DOI:** 10.1186/s40792-024-01904-y

**Published:** 2024-04-28

**Authors:** Hiroharu Shinjo, Hirono Satokawa, Hiroki Wakamatsu, Hitoshi Yokoyama

**Affiliations:** Department of Cardiovascular Surgery, Fukushima Medical University School of Medicine, Fukushima, Japan

**Keywords:** Kommerell diverticulum, Adolescence, Dysphagia

## Abstract

**Background:**

Kommerell diverticulum (KD) with right aortic arch and aberrant left subclavian artery (ALSCA) is a rare congenital aortic anomaly. To improve organ compression symptoms and avoid rupture of aneurysms in adulthood (19 years old–), surgical treatment is considered the only curative option. However, in childhood (–18 years old), several problems regarding approach and technique selection have been reported. Surgical treatment for KD in infancy (birth–2 years old) has been reported recently, but rarely in adolescence (13–19 years old). We herein report a case of KD in which the patient underwent graft replacement during adolescence.

**Case presentation:**

A 13-year-old boy was admitted to our hospital presenting with dysphagia and body weight loss. Esophagography showed upper esophageal stenosis caused by extrinsic compression. Contrast-enhanced computer tomography showed saccular aneurysm formation of KD with right aortic arch (RAA) and ALSCA. Elective surgery including KD resection and graft replacement of the descending aorta was performed via right thoracotomy under partial extracorporeal circulation. The ALSCA was reconstructed by graft interposition. No postoperative complication was observed. Follow-up esophagography showed no residual stenosis.

**Conclusion:**

We experienced a case of KD with dysphagia and weight loss in adolescence, which was successfully treated with surgery. Graft replacement could be an effective treatment option, facilitating recovery even during the growth period.

## Background

Kommerell diverticulum (KD) with right aortic arch and aberrant left subclavian artery (ALSCA) is a rare congenital aortic anomaly with an incidence of 0.05–0.1% in the general population [1]. Some KD cases have been reported, in which aneurysms developed in adulthood causing dysphagia and respiratory distress. To improve these symptoms and prevent aneurysmal rupture [2], surgery is often chosen. However, attention should be paid to the selection of approach and technique for the surgery in the period of growth. Surgical treatment for KD in infancy has been reported to date but rarely in adolescence. We herein report a case of a patient with KD who underwent graft replacement during adolescence.

## Case presentation

A 13-year-old boy (height 160.3 cm, weight 43.7 kg, BMI 17, BSA 1.419 m^2^) was admitted to our hospital presenting with dysphagia and body weight loss. He had difficulty swallowing meals and lost 3 kg in six weeks before his initial medical consultation. Blood test showed no abnormalities. Esophagography showed upper esophageal stenosis apparently caused by extrinsic compression. The patient was referred to the pediatric department of our hospital. Although his deglutition reflex was normal, he could only swallow liquids and was unable to swallow solid food.

**Fig. 1:**
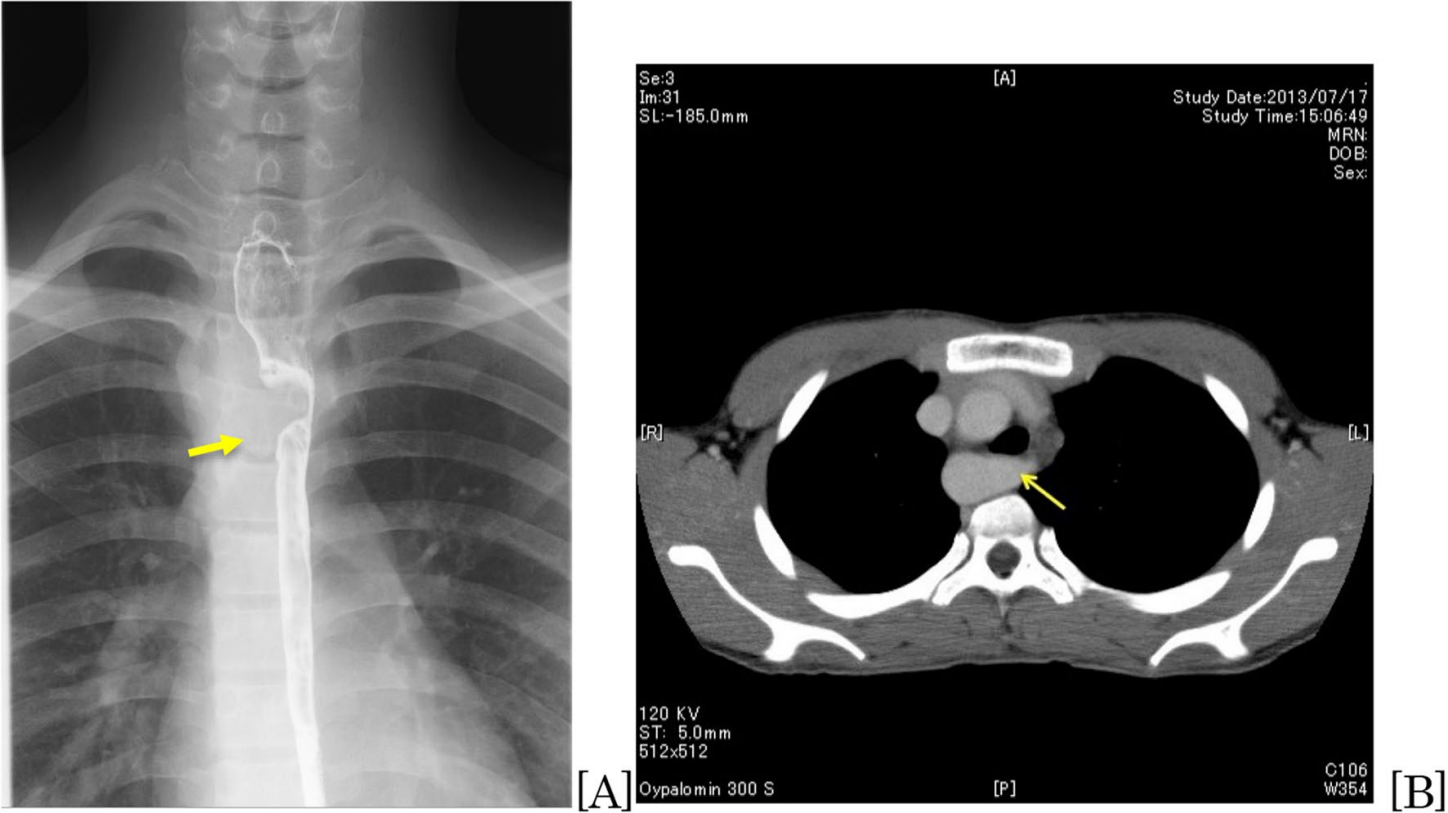
**A** Esophagography: the upper esophagus is compressed and stenotic (arrow). **B** Computer tomography: arrow shows Kommerell diverticulum (KD) and the origin of the aberrant left subclavian artery (ALSCA)

Barium esophagography showed filling defects due to compression of the upper esophagus on the right side (Fig. 1A). Cardiac echocardiography showed normal cardiac function without intracardiac anomalies. Contrast-enhanced computer tomography (CE-CT) revealed that the ALSCA with KD arose from the RAA, and the esophagus was compressed by the KD, which had a diameter of < 20 mm (Fig. 1B).

**Fig. 2:**
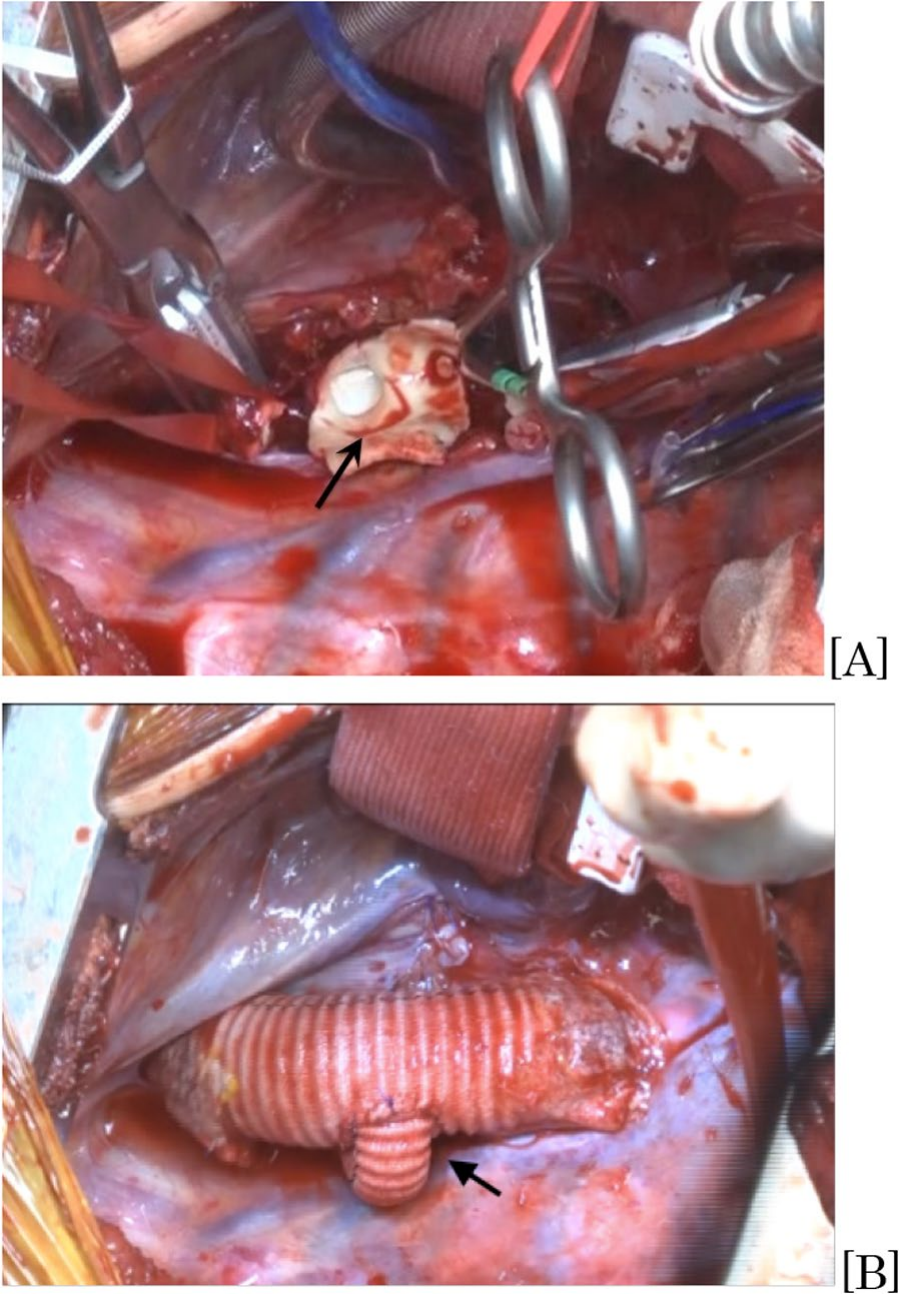
Operative findings: **A** the descending aorta is open. The KD inner surface (black arrow) and the ostium of ALSCA are visible. **B** The descending aorta is replaced using an artificial graft with the branch to the ALSCA through the dorsal aortic graft (arrow)

Elective aneurysmectomy and graft replacement of the descending aorta via right fourth intercostal thoracotomy was performed. Partial extracorporeal circulation [1.78 ~ 2.13 L/min (1.25 ~ 1.5 L/min/m^2^)] was established with drainage from the superior vena cava and perfusion to the descending aorta (Th6). The KD was exposed and clamped at the distal side of the left subclavian artery (LSA), since it was difficult to clamp the normal LSA, and resected as much as possible. The ostium of the LSA was clearly visible, and the LSA was interposed with an 8-mm graft (INTERGARD Woven, Intervascular SAS, La Ciotat, France). The descending aorta was replaced with a 20-mm graft (INTERGARD Woven, Intervascular SAS, La Ciotat, France) with bending into a slight curve to accommodate the aortic growth. The reconstructed LSA was anastomosed end-to-side on the right side through the dorsal descending aortic graft (Fig. 2). The extracorporeal circulation time was 137 min, and the operating time was 273 min. The lowest bladder temperature was 34 °C, and no blood transfusion was needed.

**Fig. 3:**
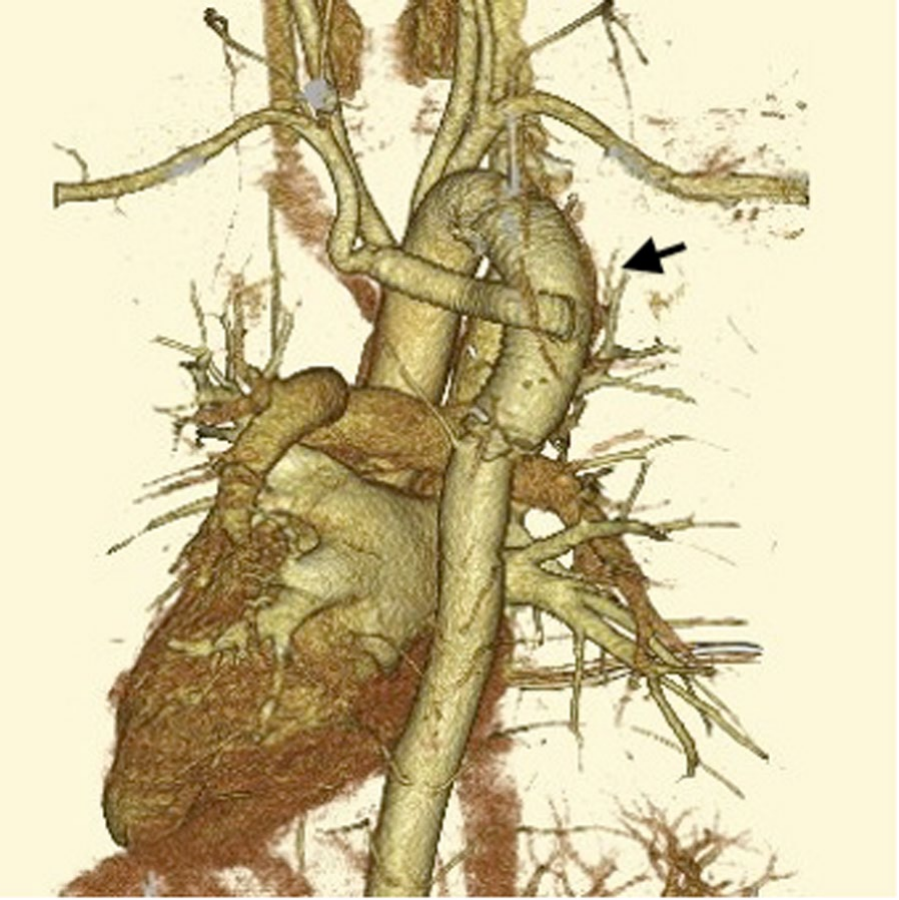
Postoperative computer tomography (posterior view): the aortic graft is slightly curved using a relatively longer graft than that of an ordinary (arrow)

The patient was extubated on the first post-operative day (POD), and was discharged from the intensive care unit on POD 2. On POD 4, he began oral intake. No dysphagia was observed postoperatively. There is no complication regarding paraplegia and phrenic or recurrent laryngeal nerve palsy. Postoperative CE-CT showed no abnormalities with reconstructed grafts (Fig. 3). He was discharged on POD 20. Barium esophagography at our outpatient clinic on POD 25 showed slight pooling of fluid. Barium was carried down smoothly by the peristalsis of the esophagus without passage disorder. The patient had no dysphagia 2 years after of surgery.

## Discussion

KD was firstly reported by Kommerell et al. in 1936 [3]. KD with RAA and ALSCA is a rare congenital aortic anomaly, with an incidence of 0.05–0.1% in the general population [1]. KD often presents with respiratory symptoms due to airway stenosis and/or dysphagia due to airway and/or esophageal compression. Emergency surgery has been reported due to rupture of an enlarged KD [4] even in asymptomatic patients. The mortality rate is 80–100% once KD is ruptured [4, 5]. Therefore, early diagnosis and surgical treatment are potentially life-saving [6].

In the current case, a KD of < 20 mm diameter caused symptoms associated with esophageal compression. In contrast, the anteroposterior diameter of the thorax (the distance from the posterior sternum to the anterior thoracic spine) at the level of the KD was < 50 mm. This relationship between the diameter of the KD and the thorax may have caused dysphasia in this case.

The indication for surgery of KD should be determined by its size and the symptoms caused by compression of the surrounding organs. Yu et al. [7] reported that surgical indications of KD in childhood is a KD diameter that is 1.5 times larger than that of the ALSCA origin, or apparent compression of the trachea caused by KD. However, there are many options depending on the case, or that there is no agreement on a standard procedure. Baker et al. [8] reported satisfactory results after performing diverticulectomy and extra-anatomical bypass from the common carotid artery to the subclavian artery bypass in 20 patients aged 9.1 ± 6.5 years old (1.5–29.1 years) with symptomatic KD. However, they reported that after each procedure, the residual diverticulum was problematic in the long-term. In addition, ligation or division of the ALSCA could cause ischemia and subclavian steal syndrome [9]. Therefore, as a curative treatment, we recommend to perform a complete resection of the diverticulum and graft replacement of the descending aorta with reconstruction of the LSA [10].

Recently, less invasive endovascular treatment of KD aneurysms has been performed in adults with low mortality and morbidity [11]. However, there are several problems with endovascular treatment during the growth period. First, the long-term durability of the endovascular approach is questionable because of the potential growth of a child’s body and vessels. The durability of the stent graft itself is also a matter of discussion because of possible material fatigue and late complications. Considering both symptomatic improvements and physical growth, we selected a more invasive procedure in the present case.

The selection of the graft size is controversial. There are few papers [12] describing the risk of aortic stenosis in adulthood due to size mismatch of the graft used in childhood. Okawa et al. [13] reported a case of acute Stanford type A aortic dissection in a 12-year-old boy who had undergone hemiarch replacement using a 22-mm graft. His descending aorta had quite a small diameter, but they used as large a graft as possible, taking his future growth into consideration.

For the diameter of the thoracic aorta, Hegde et al. [14] reported the normal ranges of effective aortic diameter in children. They reported, the effective diameter of the descending aorta was around 17 mm when the BSA was 1.4 m^2^. Even if the patient’s BSA were 2.0 m^2^, the diameter of the descending aorta would still be around 20 mm; a 20-mm graft would not cause stenosis. Therefore, in the present case, it was considered that the size of the graft selected was sufficient and allowed for the patient’s physical growth. In addition, the graft was bent with a slight curve to accommodate the aortic growth.

## Conclusion

We herein reported a case of KD with dysphagia and weight loss in an adolescent patient. The patient underwent graft replacement for KD with an uneventful postoperative course. Although surgical strategy during a patient’s adolescence is controversial, graft replacement may be a curable and effective option.

## Data Availability

Not applicable.

## Abbreviations

KD: Kommerell diverticulum
ALSCA: Aberrant left subclavian artery
AA: Right aortic arch
BMI: Body mass index
BSA: Body surface area
CE-CT: Contrast-enhanced computer tomography
LSA: Left subclavian artery
POD: Post-operative day
